# A *Caenorhabditis elegans* behavioral assay distinguishes early stage prostate cancer patient urine from controls

**DOI:** 10.1242/bio.057398

**Published:** 2021-03-26

**Authors:** Morgan Thompson, Noemi Sarabia Feria, Ally Yoshioka, Eugene Tu, Fehmi Civitci, Suzanne Estes, Josiah T. Wagner

**Affiliations:** 1 Department of Biology, Portland State University, Portland, OR 97201, USA; 2 Knight Cancer Institute Cancer Early Detection Advanced Research Center (CEDAR), Oregon Health & Science University, Portland, OR 97201, USA; 3 Molecular Genomics Laboratory, Providence St. Joseph Health, Portland, OR 97213, USA

**Keywords:** *Caenorhabditis elegans*, Nematode, Olfaction, Liquid biopsy, Prostate cancer, Volatile organic compounds

## Abstract

Current methods for non-invasive prostate cancer (PrCa) detection have a high false-positive rate and often result in unnecessary biopsies. Previous work has suggested that urinary volatile organic compound (VOC) biomarkers may be able to distinguish PrCa cases from benign disease. The behavior of the nematode *Caenorhabditis elegans* has been proposed as a tool to take advantage of these potential VOC profiles. To test the ability of *C. elegans* Bristol N2 to distinguish PrCa cases from controls, we performed chemotaxis assays using human urine samples collected from men screened for PrCa. Behavioral response of nematodes towards diluted urine from PrCa cases was compared to response to samples from cancer-free controls. Overall, we observed a significant attraction of young adult-stage *C. elegans* nematodes to 1:100 diluted urine from confirmed PrCa cases and repulsion of *C. elegans* to urine from controls. When *C. elegans* chemotaxis index was considered alongside prostate-specific antigen levels for distinguishing cancer from cancer-free controls, the accuracy of patient classification was 81%. We also observed behavioral attraction of *C. elegans* to two previously reported VOCs to be increased in PrCa patient urine. We conclude nematode behavior distinguishes PrCa case urine from controls in a dilution-dependent manner.

## Abstract (precis)

**Summary:** The nematode *Caenorhabditis elegans* shows behavioral attraction to urine from prostate cancer patients, but not to controls, and this phenomenon may be a useful tool for designing diagnostic assays or biomarker discovery.

## INTRODUCTION

Many cancers have altered metabolic pathways that support, or may even cause, malignancy (Ward and Thompson, 2012). Detection of these cancer-related metabolites or volatile compounds in blood and urine would provide an ideal means of non-invasive early cancer detection. Yet, brute-force chemical analyses have been unsuccessful in determining metabolite profiles that consistently distinguish early cancer from healthy biofluids (Liesenfeld et al., 2013). Remarkably, even in the early stages of malignancy, there is evidence that cancer patients emit odors that can be accurately detected by canine and murine olfaction (Lippi and Cervellin, 2012; Sato et al., 2017; Edwards et al., 2017; Fischer-Tenhagen et al., 2018; Elliker et al., 2014). Replicating this process of scent identification in mammals using technology or computation is hindered by the exceptionally complex neural processing in mammals that occurs to identify unique odors (Mori and Sakano, 2011). Further, mammalian detection systems rely on training and learned memory to discriminate between odors, and efficacy may be affected by animal personality, genetics, or environment (Elliker et al., 2014). Therefore, animal systems that have a naturally occurring odor discrimination for cancer patient samples could be a powerful tool for developing early cancer detection technology.

Recent work by Hirotsu et al. (2015) demonstrated the feasibility of a nematode scent detection test (NSDT) to take advantage of the chemosensory abilities of *Caenorhabditis elegans*, a small nematode worm. *C. elegans* is commonly used as an experimental model in neurobiology because of its simple nervous system, which is primarily devoted to chemosensation (Bargmann, 2006). In addition, with a sizable fraction of its genome being dedicated specifically to olfactory components, the availability of genomic tools makes *C. elegans* an appealing system for understanding sensory mechanisms (Boulin and Hobert, 2012). *C. elegans* has a well-documented ability to detect a wide variety of volatile and water-soluble compounds necessary to differentiate food, mates, pathogens and predators encountered in its natural environment (Bargmann, 2006; Bargmann et al., 1993). Using a simple assay setup, Hirotsu et al. (2015) demonstrated that *C. elegans* can detect multiple types of cancer, and potentially even pre-cancer, from 1:10 diluted urine with >95% specificity and sensitivity. This assay, known as a chemotaxis assay, is a commonly performed lab assay and has been used to score the relative attractiveness or repulsiveness of various volatile organic compounds (VOCs) to live *C. elegans* nematodes (Bargmann et al., 1993; Margie et al., 2013; Yoshida et al., 2012). Hirotsu et al. (2015) further showed that the *C. elegans* behavioral response worked strictly through G-protein coupled receptor (GPCR) mediated signaling and via chemosensory neurons known to be important for sensing VOCs. More recently and using a similar chemotaxis assay, a report by Kusumoto et al. (2020) demonstrated that *C. elegans* accurately differentiates between matched preoperative and postoperative samples from patients that underwent gastrointestinal cancer resection. Thus, the natural behavioral response of *C. elegans* to cancer patient urine may provide a useful tool for detecting cancer VOCs at early, treatable stages or for monitoring residual disease. However, the extent to which this behavioral response can effectively distinguish malignant samples from benign disease remains to be determined.

In this work, we apply a version of the NSDT to urine samples from patients with prostate cancer (PrCa) and compare the behavioral response to benign controls. We hypothesized that differentially abundant VOCs present in PrCa urine samples causes an attractive behavioral response in *C. elegans* that can distinguish them from control urine samples. The current gold-standard biomarker for non-invasive PrCa detection is prostate-specific antigen (PSA), which has high sensitivity but is known to suffer from low specificity (Thompson et al., 2004; Mistry and Cable, 2003). PSA is excreted by both malignant as well as nonmalignant epithelial cells and can therefore be present in the serum of patients with benign diseases such as prostatitis and prostatic hyperplasia (Eastham, 2017). Because overdiagnosis of PrCa often leads to invasive and risky needle biopsies (Loeb et al., 2011), biomarkers that can supplement or replace PSA are highly desired. Our findings suggest there are VOC profiles in cancer-patient urine that can distinguish early stage PrCa from control urine. This behavioral classification method appeared to be dilution dependent and yielded results that support this unique tool for developing high-throughput, non-invasive early detection screens.

## RESULTS

### Cohort clinical features

There was no significant difference among sample group means for body mass index (BMI), age at collection, or tobacco use (Table 1). Average PSA levels were determined to be significantly different among the groups (Kruskal–Wallis rank sum test, *P*<0.001) such that cancer and benign individuals had, on average, higher PSA than negative screen samples. Prostate size was nearly significantly different among groups (Kruskal–Wallis rank sum test, *P*=0.058) such that benign individuals tended to have an average larger prostate size than cancer or negative screen samples.

**Table 1.**
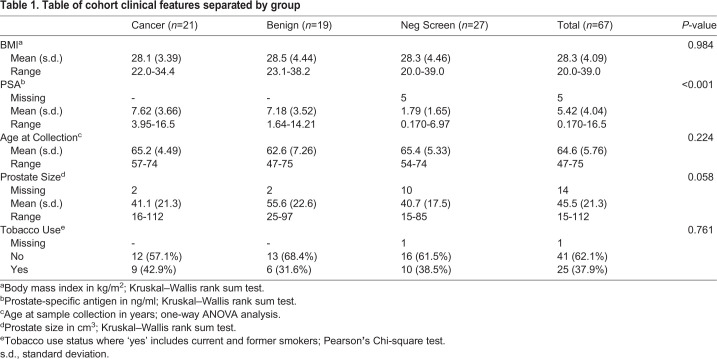
**Table of cohort clinical features separated by group**

### *C. elegans* N2 are attracted to previously identified VOC candidates for PrCa classification

**Fig. 1.**
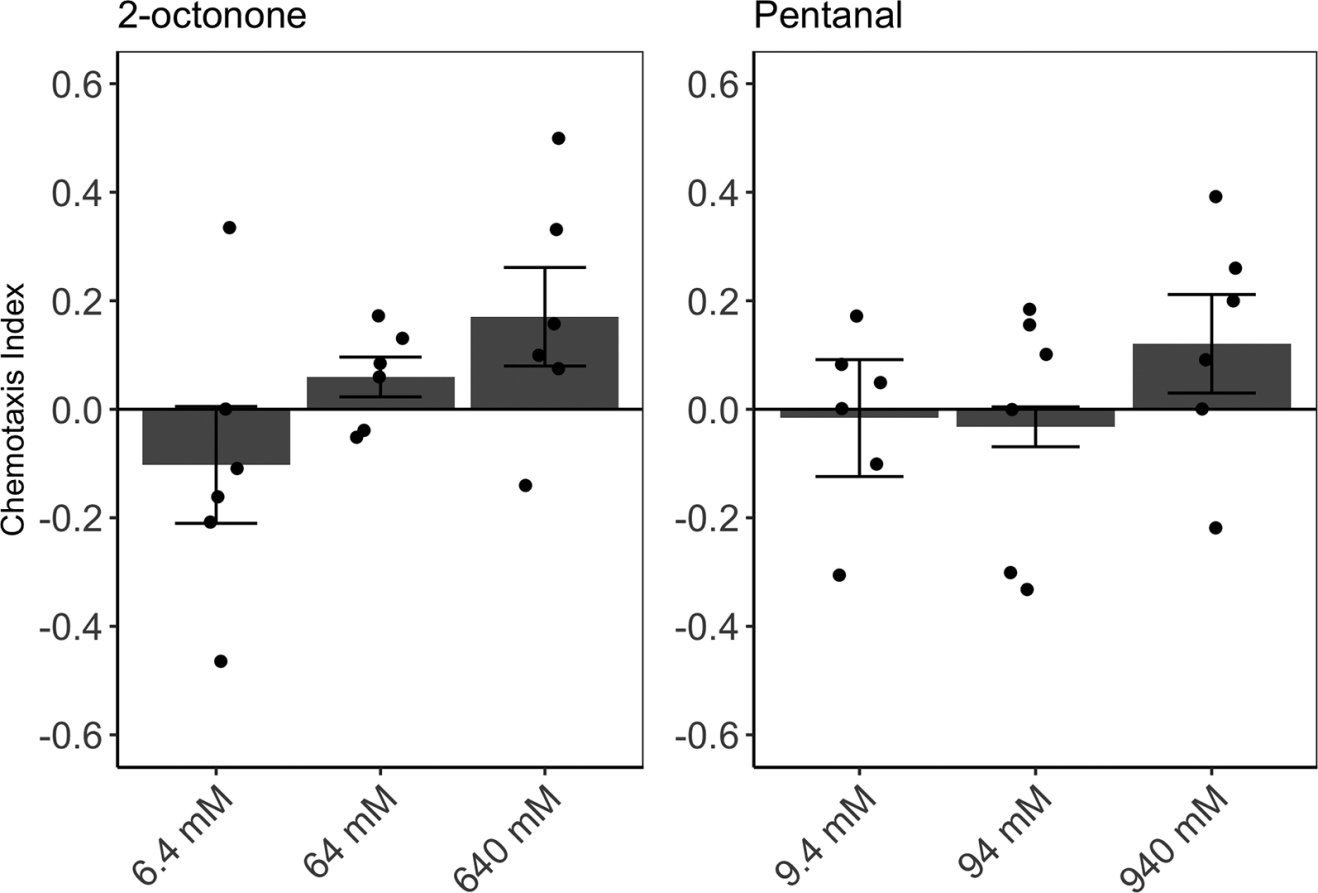
***C. elegans* N2 are attracted to two VOCs that were previously reported to be increased in prostate cancer patient urine compared to healthy controls.** Chemotaxis assays were performed using 2-octonone (left) or pentanal (right) diluted with water. *n*=6 chemotaxis assays per concentration; error bars represent mean±s.e.m..

To determine if *C. elegans* N2 displayed a behavioral response to candidate VOCs, chemotaxis assays were performed using diluted 2-octonone or pentanal as test samples. *C. elegans* N2 demonstrated overall attraction to 2-octonone at 64 mM and 640 mM (Fig. 1, left). For pentanal, *C. elegans* N2 demonstrated attraction, but only at the highest concentration of 940 mM (Fig. 1, right).

### *C. elegans* N2 nematodes demonstrate dilution-dependent behavioral response to patient urine samples

**Fig. 2.**
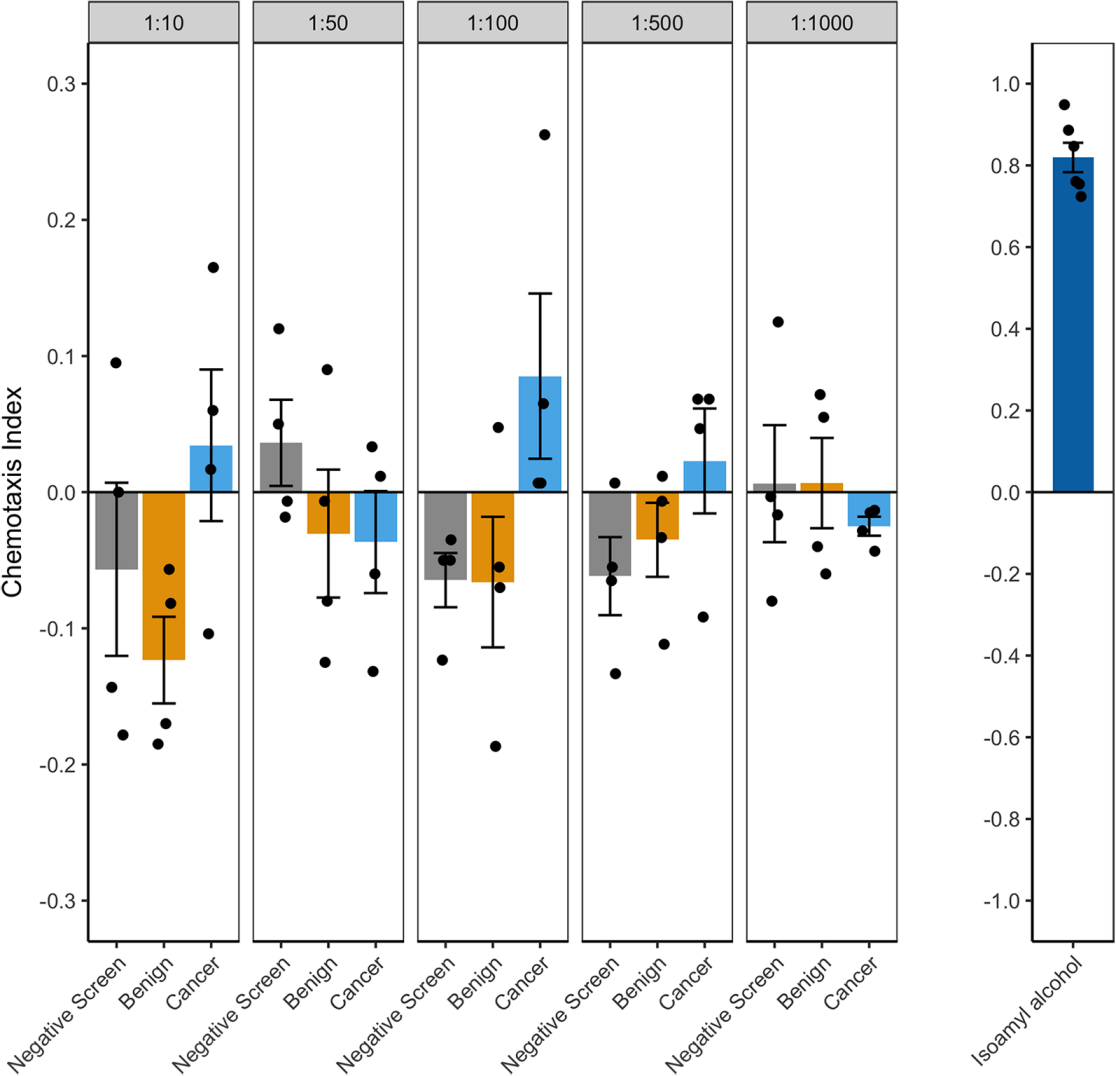
***C. elegans* N2 showed highest average attraction to 1:100 diluted PrCa patient urine compared to 1:10, 1:50, 1:500, and 1:1000 dilutions**. The same urine samples are represented across the five dilutions. Each of the three sample groups (negative screen, benign, and cancer) comprises four independent urine samples for which four to six technical replicate chemotaxis assays were performed. Data points represent the average of CI associated with each patient sample. Typical chemotaxis assay results when using 9.09 mM isoamyl alcohol as a positive control are shown on far right (*n*=6 assays). Error bars represent mean±s.e.m..

Among the five dilutions measured for chemotaxis index (CI), we observed a dilution of 1:100 to yield the best discrimination of cancer patient urine from controls in preliminary assays as defined by number of samples with overall behavioral attraction (CI >0) towards cancer samples and repulsion (CI <0) from controls (Fig. 2, left). We consistently observed high CI values in the isoamyl alcohol positive control assays (Fig. 2, right). The 1:100 urine dilution was used for all subsequent chemotaxis assays on patient samples.

### *C. elegans* N2 nematodes demonstrate an overall attraction to PrCa patient urine compared to controls

**Fig. 3.**
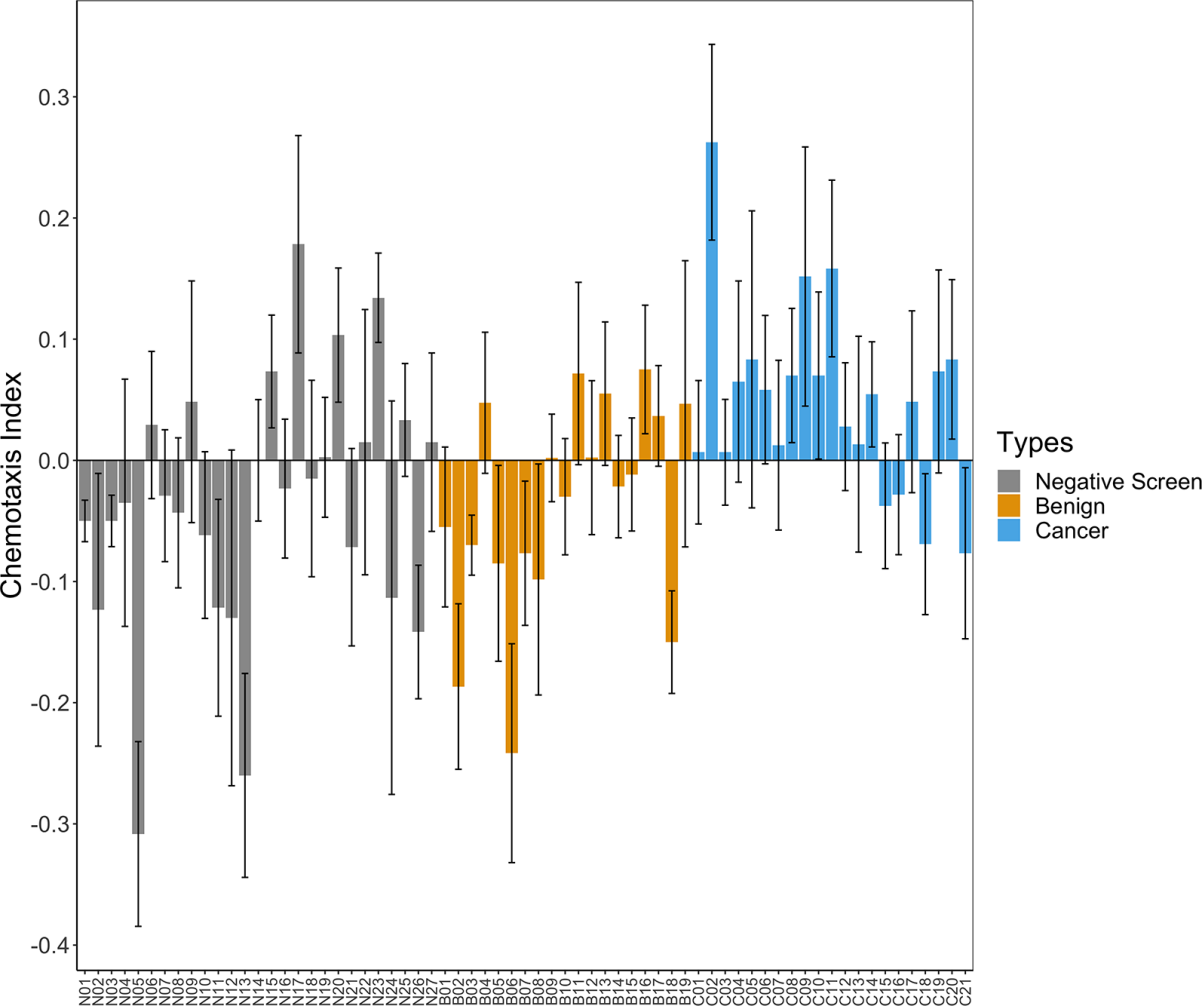
**Chemotaxis assay CIs obtained from individual patient urine sample technical replicates at 1:100 dilution.** Each bar represents average *C. elegans* N2 CIs in response to an individual patient urine sample for which four to six technical replicate chemotaxis assays were conducted. Error bars represent mean±s.e.m..

**Fig. 4.**
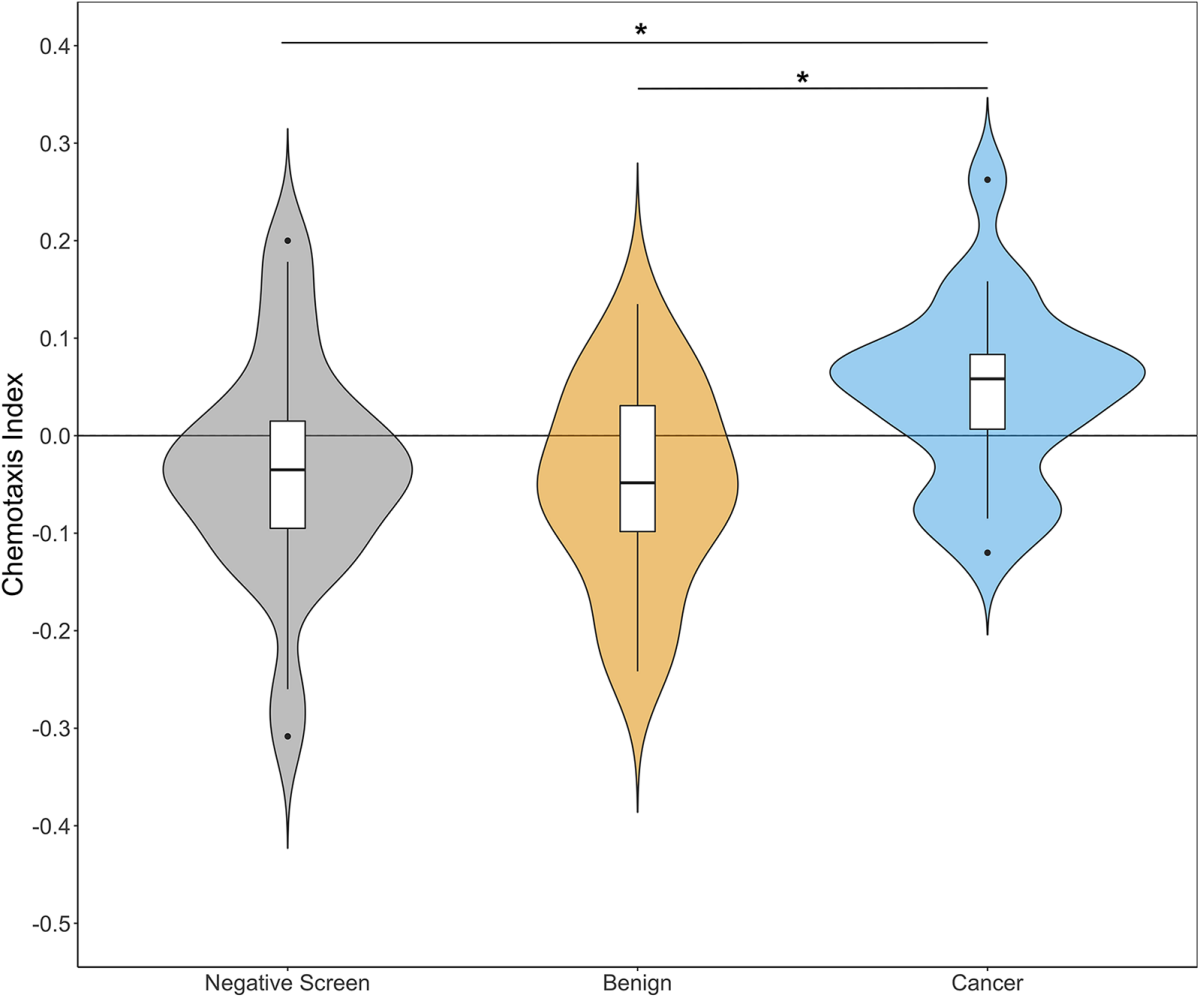
***C. elegans* N2 are more attracted to urine from PrCa patients than to benign or negative screen patient urine.** Chemotaxis assay technical replicates (*n*=4–6 assays per individual patient urine sample) were averaged for negative screen (*n*=27 individuals), benign (*n*=19 individuals), and cancer (*n*=21 individuals) patient urine samples prior to plotting and statistical testing. Boxplots for each group are inset within their respective violin plots. Dots above or below boxplots are potential outliers. Statistically different means between groups are shown with *, one-way ANOVA and Tukey’s HSD post-hoc test, *P*<0.05.

All chemotaxis assays had at least six replicates performed except for individuals N03, B04 and C02, which had four each. The average number of worms that chemotaxed in each assay for negative screen, benign and cancer groups was 37.4 (s.e.m.=1.7), 38 (s.e.m.=1.7) and 34 (s.e.m.=1.3), respectively. We observed both attractive and repulsive patient urine samples within the three groups (Fig. 3). After CIs for each patient were averaged, urine from PrCa patients elicited generally positive average CIs compared to urine from benign and negative screen individuals (Fig. 4). Shapiro–Wilk normality test results indicated the distribution of measured CIs did not significantly differ from a normal distribution for negative screen (*P*=0.45), benign (*P*=0.91) or cancer (*P*=0.95) groups. One-way statistical analysis revealed a significant difference in means among the three groups (one-way analysis of variance, ANOVA, *F*_d.f.=2_=4.71, *P*=0.012). A Tukey's honestly significant difference (HSD) post-hoc test indicated that average CIs for PrCa were significantly higher than those of benign (*P*=0.020) and negative screen (*P*=0.034), while benign and negative screen types were not significantly different (*P*=0.896). We found that PrCa urine samples CIs were significantly higher compared to benign and negative screen individuals when all technical replicates (i.e. no subsampling) were considered for each patient sample (one-way ANOVA, *F*_d.f.=2_=4.71, *P*=0.0045, Fig. S1).

### *C. elegans* N2 CI values do not significantly associate with patient clinical profile features

**Fig. 5.**
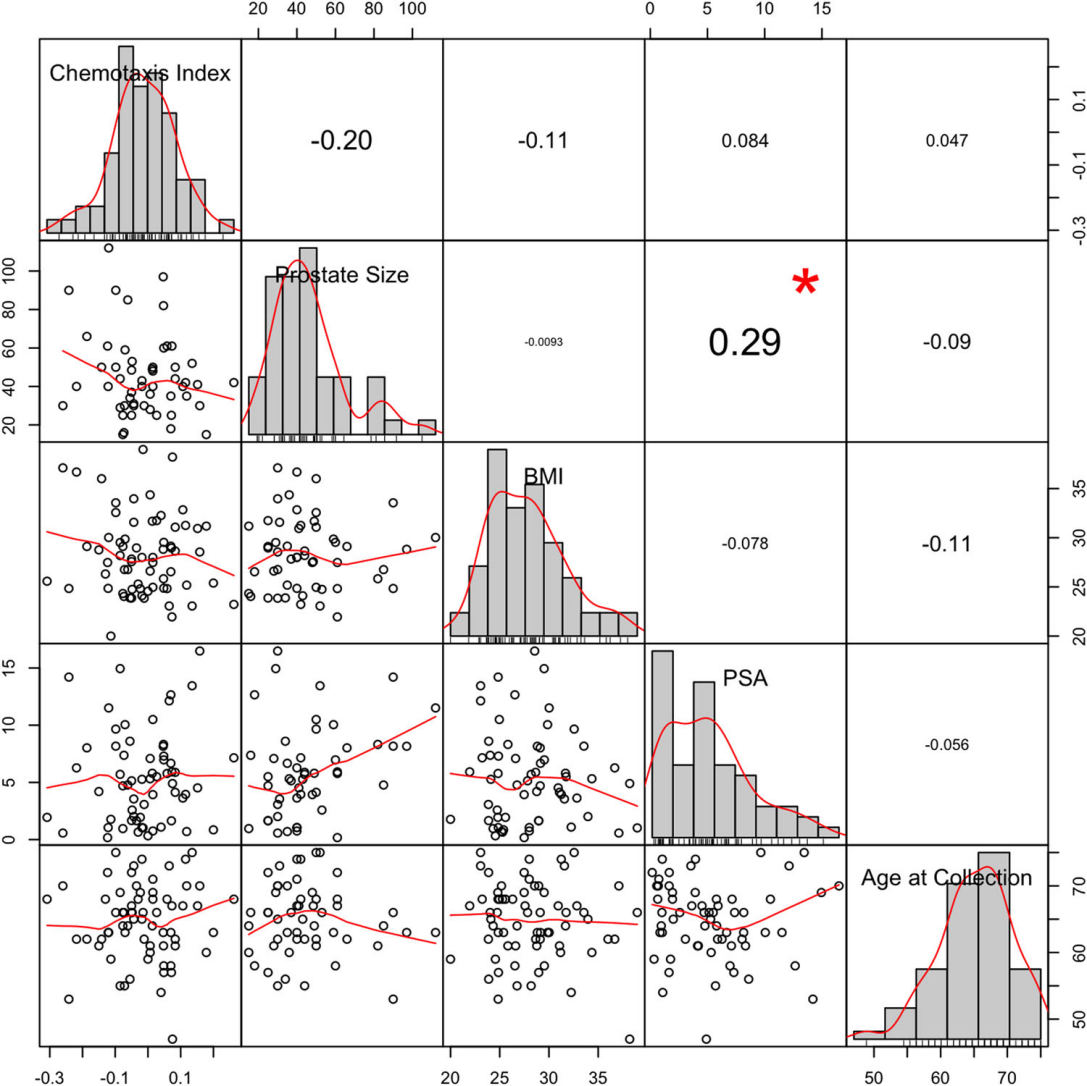
**Pearson correlation coefficients (r) comparing patient clinical features to CI.** Chemotaxis index, prostate size (cm^3^), BMI (kg/m^2^), PSA (ng/ml), and age at urine sample collection (years) for each patient were compared. Histograms include a kernel density estimation in red and rug plot. Bivariate scatter plots each have a fitted line in red. *P<0.05. BMI, body mass index; PSA, prostate-specific antigen.

We assessed potential pairwise associations between average CIs and PSA, BMI, age at collection, and prostate size. *C. elegans* N2 CIs did not correlate significantly with PSA, BMI, or age (Fig. 5). We also found no difference in average CIs between current/former smokers (*n*=25) and non-smokers (*n*=41) (Welch's two-sample *t*-test, *P*=0.81; Fig. S2, left). Similarly, we found no significant difference in the average CIs of low (3+3 or 3+4, *n*=13) and high (≥4+3, *n*=8) Gleason score tumors (Welch’s two-sample *t*-test, *P*=0.23) within the PrCa group (Fig. S2, right).

### Classification model performance of CI alone, PSA alone, and combined

To calculate sensitivity and specificity, we used classification as determined by pathology. True positives were considered to be individuals with a confirmed case of PrCa following biopsy, while true negatives were considered to be individuals who either had a negative biopsy or were deemed to not have PrCa following screening. To test the ability of average *C. elegans* N2 CIs to predict cancer status, we used a model using CI >0 to classify cancer and CI <0 to classify cancer free. With this CI model, a sensitivity of 76% and a specificity of 67% was determined for discriminating PrCa patient urine from controls. In comparison, using PSA values at the standard threshold of ≥4 ng/ml for PrCa classification yielded a sensitivity and specificity of 95% and 59%, respectively. The overall accuracy of CI for classifying PrCa versus controls was 70% (balanced accuracy=72%), slightly lower than the 71% accuracy of PSA alone (balanced accuracy=77%). Neither CI nor PSA alone were determined to have a significantly higher accuracy than the no-information rate (NIR). In addition, both CI and PSA classification models alone were determined to have significant Mcnemar's test *P*-values, and therefore both models are presumed to be fundamentally different from the classifications made by pathological assessment.

We next tested a combined classification model that required both CI >0 and PSA ≥4 ng/ml for a PrCa diagnosis and compared the model to using CI or PSA alone. Using the combined model, we were able to increase the specificity to 85% at the cost of reducing the sensitivity to 71%. The combined model accuracy improved to 81% (balanced accuracy 78%). In addition, the combined model had significantly better accuracy than the NIR of 66% (*P*=9e-3). Classification model performance comparisons are summarized in Table 2.

**Table 2.**
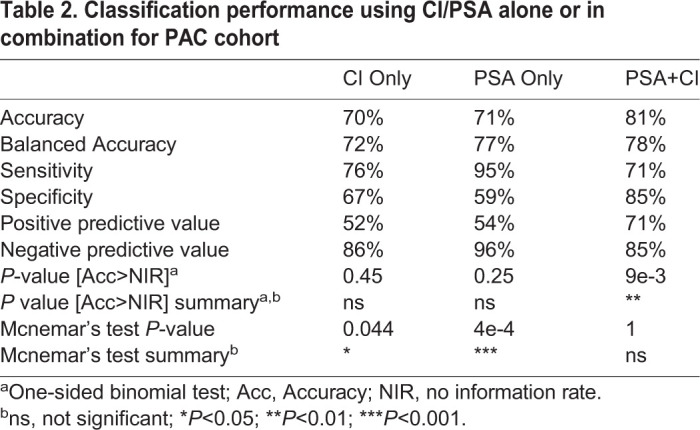
**Classification performance using CI/PSA alone or in combination for PAC cohort**

## DISCUSSION

Accumulating evidence suggests that the metabolomic profiles of patients with prostate cancer can be distinguished from normal patients and this difference could potentially be measured through blood or urine samples (Kdadra et al., 2019). The leading metabolite candidate for prostate cancer, sarcosine, has remained controversial as a specific urinary biomarker and has yet to find widespread adoption in the clinic (Sreekumar et al., 2009; Wang et al., 2018; Lima et al., 2016). Thus, the search for alternative methods of cancer metabolite biomarker discovery continues to be of great interest.

The potential for animals to sense malignancy has received increasing attention since the phenomenon was first described over three decades ago (Williams and Pembroke, 1989). Since the first report of *C. elegans* accurately classifying cancer samples by Hirotsu et al. (2015), similar methods have been applied using *C. elegans* to detect sepsis (Tee et al., 2019) and tuberculosis-specific odorants (Neto et al., 2016). Two of the VOCs proposed to be increased in PrCa urine samples by Khalid et al. (2015) were found to elicit behavioral attraction of *C. elegans* under our assay conditions. When chemotaxis assays were performed using urine samples from our cohort, our overall findings agree with the previous work by Hirotsu et al. (2015) and support a dilution-dependent behavioral response of *C. elegans* nematodes to cancer patient and control urine. It should also be noted that only one identified case of PrCa was tested in the Hirotsu et al. (2015) cohort, and thus direct comparisons of results are difficult*.* Interestingly, some urine dilutions elicited opposite behavioral responses (i.e. switching from attractive to repulsive) compared to other dilutions of the same sample. This finding was also observed by Hirotsu et al. (2015) and may be due to the complex VOC composition of human urine (Bouatra et al., 2013) causing concentration-dependent preference changes as dilutions change (Yoshida et al., 2012). Prior work by Cornu et al. (2011) reported 91% sensitivity and specificity using canine olfaction trained exclusively on urine samples from prostate cancer patients and controls. The Cornu et al. (2011) sample cohort included 33 urine samples from confirmed stages I–IV prostate cancer cases and 33 control samples from patients with negative biopsies. Our results also suggested significant olfactory discrimination of *C. elegans* to prostate cancer patient urine compared to control patients, although our observed sensitivity and specificity was lower than the results reported by Cornu et al. (2011). This reduced performance may be due in part to our cohort consisting of only early stage (stages I–II) prostate cancer samples. It is possible that there is an increase in the amount or composition of attractive VOCs as prostate cancer progresses (Kdadra et al., 2019). Our data do not indicate a clear preference by *C. elegans* for low (3+3 or 3+4) or high (≥4+3) Gleason score tumors, and the overall effect of tumor stage on animal olfaction remains to be determined.

Interestingly, our accuracy of 66% using the *C. elegans* CI for patient classification is within the range of the ∼66% accuracy reported by Khalid et al. (2015) using four VOCs. Whether any of those four VOCs are the same ones the nematodes detect in the PrCa urine samples of our cohort is unknown. Importantly, it is possible that the utility of VOCs alone in PrCa urine sample classification is limited as a biomarker. This limitation could be due to tumor heterogeneity, wherein only some prostate adenocarcinomas produce the identifying VOC(s), or high among-individual variation in VOC expression that can mask signal. Importantly, however, our data suggest that the *C. elegans* CI in response to urine samples is independent of blood-derived PSA and combining the two measurements increased the accuracy of the classification model to 81%. A similar finding was reported by Khalid et al. (2015) and later by Gao et al. (2019) whereby the sensitivity and specificity of PrCa VOC profiling by GC-MS was significantly improved by also using PSA. Thus, our study further supports supplementing PSA models with VOC profiles as a means to increase the accuracy of PrCa detection.

Because knowledge of the specific ligand-GPCR interactions that determine *C. elegans* chemotactic behavior remains limited (Bargmann, 2006; Tobin, 2008; Vidal et al., 2018), the exact olfactory receptors that may be responsible for *C. elegans* behavior-based cancer detection are unknown. Regardless of this limitation, there are approaches that could be further developed to leverage *C. elegans* olfaction as a diagnostic or biomarker discovery tool. The first approach is to develop high-throughput and reproducible technology that can use the animal behavior preference itself as a diagnostic assay (e.g. the ‘N-nose’ demonstrated by Hirotsu et al., 2015 and Kusumoto et al., 2020). Another possibility is to use the behavioral assay as an unbiased sensor system that could be coupled with a discovery method such as GC-MS. In this method, the behavioral assay has a similar function to an ‘electronic nose’, but with a potentially increased limit of detection and no need for machine learning (de Boer et al., 2014). A previous study in the moth *Manduca sexta* recorded the neural responses of *M. sexta* to identify complex odors of interest fractionated by GC (Riffell et al., 2009). More recently, work by Trivedi et al. (2019) leveraged the remarkable olfactory ability of a human ‘super smeller’ to annotate GC-MS spectra for biomarker discovery in Parkinson's disease patient sebum, the lipid secretions of sebaceous glands in the skin. Trivedi et al. (2019) concluded that VOC profiles in Parkinson's disease patient sebum could not be accurately distinguished from normal controls when relying solely on unsupervised clustering. Combined with the extensive repertoire of genetic and neural biology tools available to the *C. elegans* model system, our data support the possibility of using *C. elegans* olfaction for biomarker discovery in highly heterogeneous diseases where among-sample variation otherwise makes unsupervised learning difficult.

Biomarkers that can be obtained noninvasively and boost the accuracy of PSA for PrCa detection are highly desired. In this work, *C. elegans* demonstrated a weak but significant attraction to urine from PrCa patients when measured by behavioral assay. The *C. elegans* behavioral assay did not misclassify the same patients as PSA and combining the two outcomes increased overall accuracy and specificity. The potentially independent value of CI, and thus potentially of VOCs of interest, was further supported by the lack of CI correlation with other clinical attributes. While we were unable to determine the VOCs that elicited the behavioral response, our results support previous work that animal olfactory responses could be a useful tool for cancer biomarker discovery.

## MATERIALS AND METHODS

### Subject inclusion criteria and urine sample collection

All experimental protocols were reviewed and approved by the Oregon Health and Science University Institutional Review Board (protocol number 18048). Informed consent was obtained from all patients included in this study. Urine from human subjects was acquired based on the following inclusion criteria: (1) male patients between 45–75 years old at time of consent, (2) no current or previous cancer diagnosis (excluding squamous cell/basal cell carcinoma), (3) a total PSA between 2.5–20 ng/ml drawn within the last 2 years, (4) no more than one previous negative biopsy, (5) no prostate MRI with Pi-RADS four or five lesions, (6) no digital rectal exam (DRE) score of cT3/4 (bilateral nodules), and (7) no history of prostate intervention within the last 6 months. Patient urine samples were collected at the Oregon Health and Science University and Portland Veterans Affairs Medical Center urology clinics. Urine samples were collected uniformly in preservative-free collection cups, transported at 4°C, and stored at -80°C. All urine samples were collected post-DRE and prior to any biopsy or surgical resection (if applicable). The cohort included urine samples from PrCa cases, control benign patients, and control negative screen patients. For each patient, we received one urine sample and therefore define each urine sample as a separate biological replicate. For the PrCa urine samples, patients were confirmed to have prostate adenocarcinoma (stage I or II) by a pathologist following biopsy and/or surgical resection. Gleason scores were also determined for positive PrCa cases. We categorized Gleason scores as having either low (Gleason score 3+3 or 3+4) or high (Gleason score ≥4+3) probability of postoperative progression (Chan et al., 2000). For benign urine samples, patients were suspected to have prostate carcinoma, but biopsy results did not have evidence for carcinoma. Patients with benign diagnosis may have had other pathologies or atypical results that are not cancer. For negative screen urine samples, patients presented urinary tract symptoms or were undergoing routine screening, but following screening examination and DRE, a prostate biopsy or fine-needle aspiration was deemed not necessary and was therefore not performed. The final study cohort included 21 urine samples from confirmed PrCa cases, 19 urine samples from control benign patients, and 27 urine samples from control negative screen patients (Table 1).

### Nematode strains and culture conditions

*C. elegans* Bristol N2 (referred to as ‘N2’) was used for all chemotaxis assays. *C. elegans* N2, a commonly used lab strain, has experienced many generations of lab culture before being cryogenically stored as separate strain in 1980 (WormBase). Following Hirotsu et al. (2015), nematodes were maintained at 20°C on Nematode Growth Medium Lite (NGML; US Biological, N1005) plates seeded with a lawn of NA22 *Escherichia coli* as a food source.

### Nematode age synchronization

Prior to all experiments, strains were allowed to recover from freezing for two to three generations prior to use. In accordance with Hirotsu et al. (2015), young adult stage nematodes were used for all chemotaxis assays. All centrifugation steps were performed at room temperature (i.e. 21°C). Mixed-age nematode populations were age synchronized using standard bleach methods (Stiernagle, 1999). The resulting egg pellet was then resuspended into the 1 ml of remaining buffer solution and the egg–buffer solution was pipetted onto new NGML plates seeded with NA22 *E. coli.* The plated eggs were maintained at 20°C for 72 h to allow age-synchronized nematodes to hatch and reach the young adult stage.

### Chemotaxis assays and calculation of chemotaxis index

**Fig. 6.**
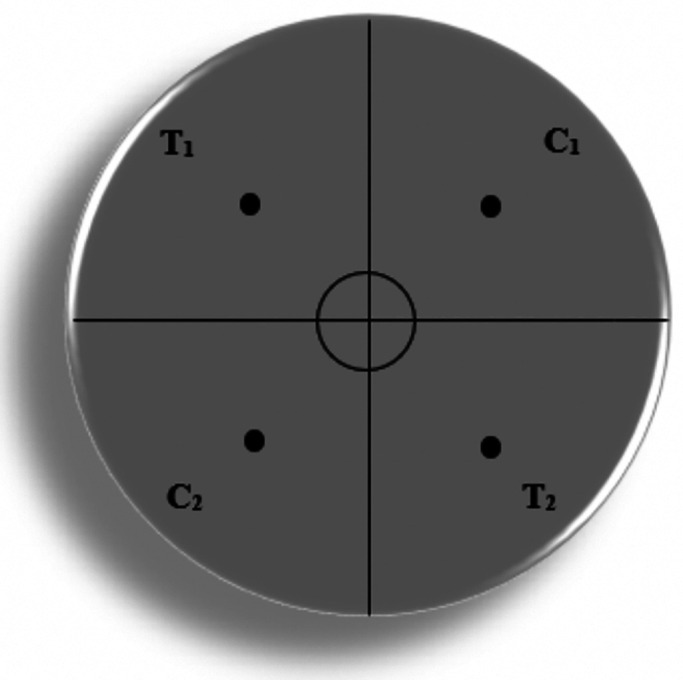
**Chemotaxis assay plate setup.** Diluted sample was placed on the black dots in T1 and T2 quadrants. Sample diluent (water) was placed on the black dots in C1 and C2 quadrants. Approximately 50 young adult stage nematodes were placed in the center circle and allowed to migrate for 1 h. The plate was then photographed and nematodes in each quadrant were recorded.

We used a chemotaxis assay plate format with diagonally opposing sample quadrants to reduce the potential of random worm movements affecting results. Following Margie et al. (2013), unseeded NGML plates prepared without antibiotic were divided into four equally sized quadrants (Fig. 6). A circle with a diameter of 1 cm was drawn around the center of the plate. Quadrants across from one another were labelled with ‘T’ for the sample (previously reported VOC or diluted urine) and the other two quadrants were labelled ‘C’ for control diluent (water). Each quadrant contained a dot 2.5 cm from the center of the plate for placement of 1 µl of sample or water. Approximately 50 washed, young adult nematodes were plated in the center of the assay plate. We controlled for light by using clean, unscented towels to cover the chemotaxis plates during assays. The plates were left on the benchtop, covered with towels for 1 h at room temperature (21°C). Assays were performed for 1 h at room temperature to match the length and temperature conditions of the chemotaxis assays performed by Hirotsu et al. (2015). After 1 h, the plates were individually photographed and the number of nematodes in each quadrant was recorded. Each completed chemotaxis assay was considered a technical replicate for statistical analysis. An example completed chemotaxis assay is shown in Fig. S3.

To calculate an overall score of behavioral response to a sample, hereafter referred to as the chemotaxis index (CI), the equation:(1)
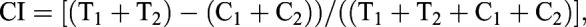
was used where T_1_+T_2_ was the number of nematodes that migrated into the first and second quadrants containing the sample being tested and C_1_+C_2_ is the number of nematodes that migrated into the first and second quadrants containing only water, respectively (Margie et al., 2013). To control for nematodes that were immobile or incapable of olfaction, all animals in the 1 cm circle near the center were not counted. Thus, a positive CI when calculated using Eqn 1 indicates nematode attraction towards a sample, a negative CI indicates repulsion from a sample, and a CI=0 indicates no behavioral preference. We define a technical replicate as a single completed chemotaxis assay using one diluted sample. At least 4–6 technical replicates were performed for each urine sample or VOC compound.

### Chemotaxis assays on previously reported PrCa VOCs

CIs were obtained for two VOCs previously identified by Khalid et al. (2015) to be potential biomarkers of PrCa. Chemotaxis assays were performed using serial dilutions of 2-octonone (6.4 mM, 64 mM and 640 mM; Sigma-Aldrich 2479, analytical standard grade, ≥99.5% purity) or pentanal (9.4 mM, 94 mM, 940 mM; Sigma-Aldrich 42272, analytical standard grade, ≥97.5% purity). Six replicates of each dilution prepared in water were performed identically to the urine chemotaxis assays.

### Chemotaxis assays on patient urine samples

Prior to use in chemotaxis assays, urine samples were thawed at room temperature and inverted three times before being freshly diluted in sterile Milli-Q filtered water. The same diluted sample was used for all replicates performed on the same day. We first determined the optimal urine dilution that can distinguish cancer from controls by performing chemotaxis assays on serially diluted urine at 1:10, 1:50, 1:100, 1:500, and 1:1000 dilutions. For these assays, the same four patients per group were used for all five dilutions. We considered the best urine dilution to have the highest overall accuracy rate for discriminating cancer versus control samples, based on a positive or negative CI, and the highest mean difference between CI when comparing cancer and control groups. Following determination of the best urine dilution, we performed chemotaxis assays for the remaining urine samples only at that dilution. Each round of chemotaxis assays included six samples assayed at the same time: one unblinded sample from each of the three groups (negative screen, benign, and cancer) and three samples with blinded disease status. The disease status of the blinded samples was revealed following completion of all assays. In some cases, more than six chemotaxis assay technical replicates were performed in total for some unblinded samples. To prevent skewed oversampling of these individuals, six CI technical replicates were randomly sampled and those six CIs were used to calculate the average CIs for the individuals. Therefore, four to six chemotaxis assay CIs are averaged to calculate one CI per patient sample (i.e. biological replicate). As a positive control, at least three chemotaxis assay replicates using the known attractant isoamyl alcohol (TCI, I0289, >99.0% purity) as a sample were performed alongside urine sample assays for each assay block (Bargmann et al., 1993). The isoamyl alcohol was diluted to 9.09 mM prior to use.

### Data analysis

Statistical analysis, subsampling, and plot generation was conducted using R (v.3.6.1) and Rstudio (v.1.2.5019). For each patient sample, CI replicates were averaged prior to statistical analysis. CI and clinical data were tested for normality using the Shapiro–Wilk test with the null hypothesis that the data are normally distributed (parametric). Differences in nematode chemotactic response to urine types (negative screen, benign disease, or prostate cancer) were determined using a one-way ANOVA. Following a statistically significant one-way ANOVA, pairwise differences between group means were determined post hoc using Tukey's HSD test (α=0.05). To test for differences in CI means between two groups categorized by clinical data, a Welch two sample *t*-test was used. For clinical attribute comparisons between groups, one-way ANOVAs were applied to parametric clinical data, the Kruskal–Wallis rank sum test was applied to non-parametric clinical data, and Pearson's Chi-square test was applied to categorical. Classification model performance was evaluated using the coin R package and default parameters (Hothorn et al., 2006). Correlations and line fitting using clinical data were performed with the PerformanceAnalytics R package (Peterson et al., 2014). *P*-values were adjusted for multiple comparisons and statistical significance was determined at *P*<0.05 for all tests. Plots were generated using the ggplot2 R package (Wickham, 2016) For boxplots, potential outliers were determined using the default parameters in the ggplot2 package.

## Supplementary Material

Supplementary information

## References

[BIO057398C1] Bargmann, C. I. (2006). Chemosensation in *C. elegans*. WormBook, ed. The *C. elegans* Research Community, WormBook. 10.1895/wormbook.1.123.1, http://www.wormbook.org.

[BIO057398C2] Bargmann, C. I., Hartwieg, E. and Horvitz, H. R. (1993). Odorant-selective genes and neurons mediate olfaction in *C. elegans*. *Cell* 74, 515-527. 10.1016/0092-8674(93)80053-H 8348618

[BIO057398C3] Bouatra, S., Aziat, F., Mandal, R., Guo, A. C., Wilson, M. R., Knox, C., Bjorndahl, T. C., Krishnamurthy, R., Saleem, F., Liu, P. et al. (2013). The human urine metabolome. *PloS ONE* 8, e73076. 10.1371/journal.pone.0073076 24023812 PMC3762851

[BIO057398C4] Boulin, T. and Hobert, O. (2012). From genes to function: the C. elegans genetic toolbox. *Wiley Interdiscip. Rev. Dev. Biol.* 1, 114-137. 10.1002/wdev.1 23801671 PMC3694748

[BIO057398C5] Chan, T. Y., Partin, A. W., Walsh, P. C. and Epstein, J. I. (2000). Prognostic significance of Gleason score 3+4 versus Gleason score 4+3 tumor at radical prostatectomy. *Urology* 56, 823-827. 10.1016/S0090-4295(00)00753-6 11068310

[BIO057398C6] Cornu, J.-N., Cancel-Tassin, G., Ondet, V., Girardet, C. and Cussenot, O. (2011). Olfactory detection of prostate cancer by dogs sniffing urine: a step forward in early diagnosis. *Eur. Urol.* 59, 197-201. 10.1016/j.eururo.2010.10.006 20970246

[BIO057398C7] de Boer, N. K. H., de Meij, T. G. J., Oort, F. A., Ben Larbi, I., Mulder, C. J. J., van Bodegraven, A. A. and van der Schee, M. P. (2014). The scent of colorectal cancer: detection by volatile organic compound analysis. *Clin. Gastroenterol. Hepatol.* 12, 1085-1089. 10.1016/j.cgh.2014.05.005 24823289

[BIO057398C8] Eastham, J. (2017). Prostate cancer screening. *Invest. Clin. Urol.* 58, 217-219. 10.4111/icu.2017.58.4.217 PMC5494343 28681029

[BIO057398C9] Edwards, T. L., Browne, C. M., Schoon, A., Cox, C. and Poling, A. (2017). Animal olfactory detection of human diseases: guidelines and systematic review. *J. Vet. Behav.* 20, 59-73. 10.1016/j.jveb.2017.05.002

[BIO057398C10] Elliker, K. R., Sommerville, B. A., Broom, D. M., Neal, D. E., Armstrong, S. and Williams, H. C. (2014). Key considerations for the experimental training and evaluation of cancer odour detection dogs: lessons learnt from a double-blind, controlled trial of prostate cancer detection. *BMC Urol.* 14, 22. 10.1186/1471-2490-14-22 24575737 PMC3945616

[BIO057398C11] Fischer-Tenhagen, C., Johnen, D., Nehls, I. and Becker, R. (2018). A Proof of concept: are detection dogs a useful tool to verify potential biomarkers for lung cancer? *Front. Vet. Sci.* 5, 52. 10.3389/fvets.2018.00052 29594162 PMC5861141

[BIO057398C12] Gao, Q., Su, X., Annabi, M. H., Schreiter, B. R., Prince, T., Ackerman, A., Morgas, S., Mata, V., Williams, H. and Lee, W.-Y. (2019). Application of urinary volatile organic compounds (VOCs) for the diagnosis of prostate cancer. *Clin. Genitourin Cancer* 17, 183-190. 10.1016/j.clgc.2019.02.003 30853355

[BIO057398C13] Hirotsu, T., Sonoda, H., Uozumi, T., Shinden, Y., Mimori, K., Maehara, Y., Ueda, N. and Hamakawa, M. (2015). A highly accurate inclusive cancer screening test using Caenorhabditis elegans scent detection. *PloS ONE* 10, e0118699. 10.1371/journal.pone.0118699 25760772 PMC4356513

[BIO057398C14] Hothorn, T., Hornik, K., Van De Wiel, M. A. and Zeileis, A. (2006). A lego system for conditional inference. *Am. Statistician* 60, 257-263. 10.1198/000313006X118430

[BIO057398C15] Kdadra, M., Höckner, S., Leung, H., Kremer, W. and Schiffer, E. (2019). Metabolomics biomarkers of prostate cancer: a systematic review. *Diagnostics* 9, 21. 10.3390/diagnostics9010021 PMC6468767 30791464

[BIO057398C16] Khalid, T., Aggio, R., White, P., De Lacy Costello, B., Persad, R., Al-Kateb, H., Jones, P., Probert, C. S. and Ratcliffe, N. (2015). Urinary volatile organic compounds for the detection of prostate cancer. *PloS ONE* 10, e0143283. 10.1371/journal.pone.0143283 26599280 PMC4657998

[BIO057398C17] Kusumoto, H., Tashiro, K., Shimaoka, S., Tsukasa, K., Baba, Y., Furukawa, S., Furukawa, J., Niihara, T., Hirotsu, T. and Uozumi, T. (2020). Efficiency of gastrointestinal cancer detection by Nematode-NOSE (N-NOSE). *In Vivo* 34, 73-80. 10.21873/invivo.11747 31882465 PMC6984105

[BIO057398C18] Liesenfeld, D. B., Habermann, N., Owen, R. W., Scalbert, A. and Ulrich, C. M. (2013). Review of mass spectrometry–based metabolomics in cancer research. *Cancer Epidemiol. Biomark. Prev.* 22, 2182-2201. 10.1158/1055-9965.EPI-13-0584 PMC3912559 24096148

[BIO057398C19] Lima, A. R., de Lourdes Bastos, M., Carvalho, M. and de Pinho, P. G. (2016). Biomarker discovery in human prostate cancer: an update in metabolomics studies. *Transl. Oncol.* 9, 357-370. 10.1016/j.tranon.2016.05.004 27567960 PMC5006818

[BIO057398C20] Lippi, G. and Cervellin, G. (2012). Canine olfactory detection of cancer versus laboratory testing: myth or opportunity? *Clin. Chem. Lab. Med.* 50, 435-439. 10.1515/cclm.2011.672 21790506

[BIO057398C21] Loeb, S., Carter, H. B., Berndt, S. I., Ricker, W. and Schaeffer, E. M. (2011). Complications after prostate biopsy: data from SEER-medicare. *J. Urol.* 186, 1830-1834. 10.1016/j.juro.2011.06.057 21944136 PMC9840843

[BIO057398C22] Margie, O., Palmer, C. and Chin-Sang, I. (2013). C. elegans chemotaxis assay. *J. Vis. Exp.* 74, e50069. 10.3791/50069 PMC3667641 23644543

[BIO057398C23] Mistry, K. and Cable, G. (2003). Meta-analysis of prostate-specific antigen and digital rectal examination as screening tests for prostate carcinoma. *J. Am. Board Fam. Med.* 16, 95-101. 10.3122/jabfm.16.2.95 12665174

[BIO057398C24] Mori, K. and Sakano, H. (2011). How is the olfactory map formed and interpreted in the mammalian brain? *Annu. Rev. Neurosci.* 34, 467-499. 10.1146/annurev-neuro-112210-112917 21469960

[BIO057398C25] Neto, M. F., Nguyen, Q. H., Marsili, J., McFall, S. M. and Voisine, C. (2016). The nematode Caenorhabditis elegans displays a chemotaxis behavior to tuberculosis-specific odorants. *J. Clin. Tuberc. Other Mycobacterial Dis.* 4, 44-49. 10.1016/j.jctube.2016.06.001 PMC6850256 31723687

[BIO057398C26] Peterson, B. G., Carl, P., Boudt, K., Bennett, R., Ulrich, J. and Zivot, E. (2014). PerformanceAnalytics: econometric tools for performance and risk analysis. R package version 1.3.

[BIO057398C27] Riffell, J. A., Lei, H. and Hildebrand, J. G. (2009). Neural correlates of behavior in the moth Manduca sexta in response to complex odors. *Proc. Natl. Acad. Sci. USA* 106, 19219-19226. 10.1073/pnas.0910592106 19907000 PMC2780752

[BIO057398C28] Sato, T., Katsuoka, Y., Yoneda, K., Nonomura, M., Uchimoto, S., Kobayakawa, R., Kobayakawa, K. and Mizutani, Y. (2017). Sniffer mice discriminate urine odours of patients with bladder cancer: a proof-of-principle study for non-invasive diagnosis of cancer-induced odours. *Sci. Rep.* 7, 14628. 10.1038/s41598-017-15355-z 29116175 PMC5676727

[BIO057398C29] Sreekumar, A., Poisson, L. M., Rajendiran, T. M., Khan, A. P., Cao, Q., Yu, J., Laxman, B., Mehra, R., Lonigro, R. J., Li, Y. et al. (2009). Metabolomic profiles delineate potential role for sarcosine in prostate cancer progression. *Nature* 457, 910. 10.1038/nature07762 19212411 PMC2724746

[BIO057398C30] Stiernagle, T. (1999). Maintenance of *C. elegans*. *C. elegans*: A Practical Approach (ed. I. Hope), pp. 51-67.

[BIO057398C31] Tee, L. F., Tan, T. L., Neoh, H.-M. and Jamal, R. (2019). Rapid detection of sepsis using CESDA: the Caenorhabditis elegans Sepsis detection assay. *Rev. Soc. Bras. Med. Trop.* 52, e20180300. 10.1590/0037-8682-0300-2018 30892548

[BIO057398C32] Thompson, I. M., Pauler, D. K., Goodman, P. J., Tangen, C. M., Lucia, M. S., Parnes, H. L., Minasian, L. M., Ford, L. G., Lippman, S. M., Crawford, E. D. et al. (2004). Prevalence of prostate cancer among men with a prostate-specific antigen level ≤4.0 ng per milliliter. *N. Engl. J. Med.* 350, 2239-2246. 10.1056/NEJMoa031918 15163773

[BIO057398C33] Tobin, A. B. (2008). G-protein-coupled receptor phosphorylation: where, when and by whom. *Br. J. Pharmacol.* 153, S167-SS76. 10.1038/sj.bjp.0707662 18193069 PMC2268057

[BIO057398C34] Trivedi, D. K., Sinclair, E., Xu, Y., Sarkar, D., Walton-Doyle, C., Liscio, C., Banks, P., Milne, J., Silverdale, M., Kunath, T. et al. (2019). Discovery of volatile biomarkers of Parkinson's disease from sebum. *ACS Cent. Sci.* 5, 599-606. 10.1021/acscentsci.8b00879 31041379 PMC6487537

[BIO057398C35] Vidal, B., Aghayeva, U., Sun, H., Wang, C., Glenwinkel, L., Bayer, E. A. and Hobert, O. (2018). An atlas of Caenorhabditis elegans chemoreceptor expression. *PLoS Biol.* 16, e2004218. 10.1371/journal.pbio.2004218 29293491 PMC5749674

[BIO057398C36] Wang, M., Zou, L., Liang, J., Wang, X., Zhang, D., Fang, Y., Zhang, J., Xiao, F. and Liu, M. (2018). The urinary sarcosine/creatinine ratio is a potential diagnostic and prognostic marker in prostate cancer. *Med. Sci. Monit.* 24, 3034-3041. 10.12659/MSM.909949 29741162 PMC5967288

[BIO057398C37] Ward, P. S. and Thompson, C. B. (2012). Metabolic reprogramming: a cancer hallmark even warburg did not anticipate. *Cancer Cell* 21, 297-308. 10.1016/j.ccr.2012.02.014 22439925 PMC3311998

[BIO057398C38] Wickham, H. (2016). *ggplot2: Elegant Graphics for Data Analysis*. Springer. https://link.springer.com/book/10.1007%2F978-0-387-98141-3

[BIO057398C39] Williams, H. and Pembroke, A. (1989). Sniffer dogs in the melanoma clinic? *Lancet* 333, 734. 10.1016/S0140-6736(89)92257-5 2564551

[BIO057398C40] Yoshida, K., Hirotsu, T., Tagawa, T., Oda, S., Wakabayashi, T., Iino, Y. and Ishihara, T. (2012). Odour concentration-dependent olfactory preference change in *C. elegans*. *Nat. Commun.* 3, 739. 10.1038/ncomms1750 22415830

