# Supplementary material for: A Caenorhabditis elegans behavioral assay distinguishes early stage prostate cancer patient urine from controls
Source: Biol Open. 2021 Mar 26;10(3):bio057398. doi: 10.1242/bio.057398 (PMC8015240; doi:10.1242/bio.057398)
Supplement: Supplementary information [file biolopen-10-057398-s1.pdf]

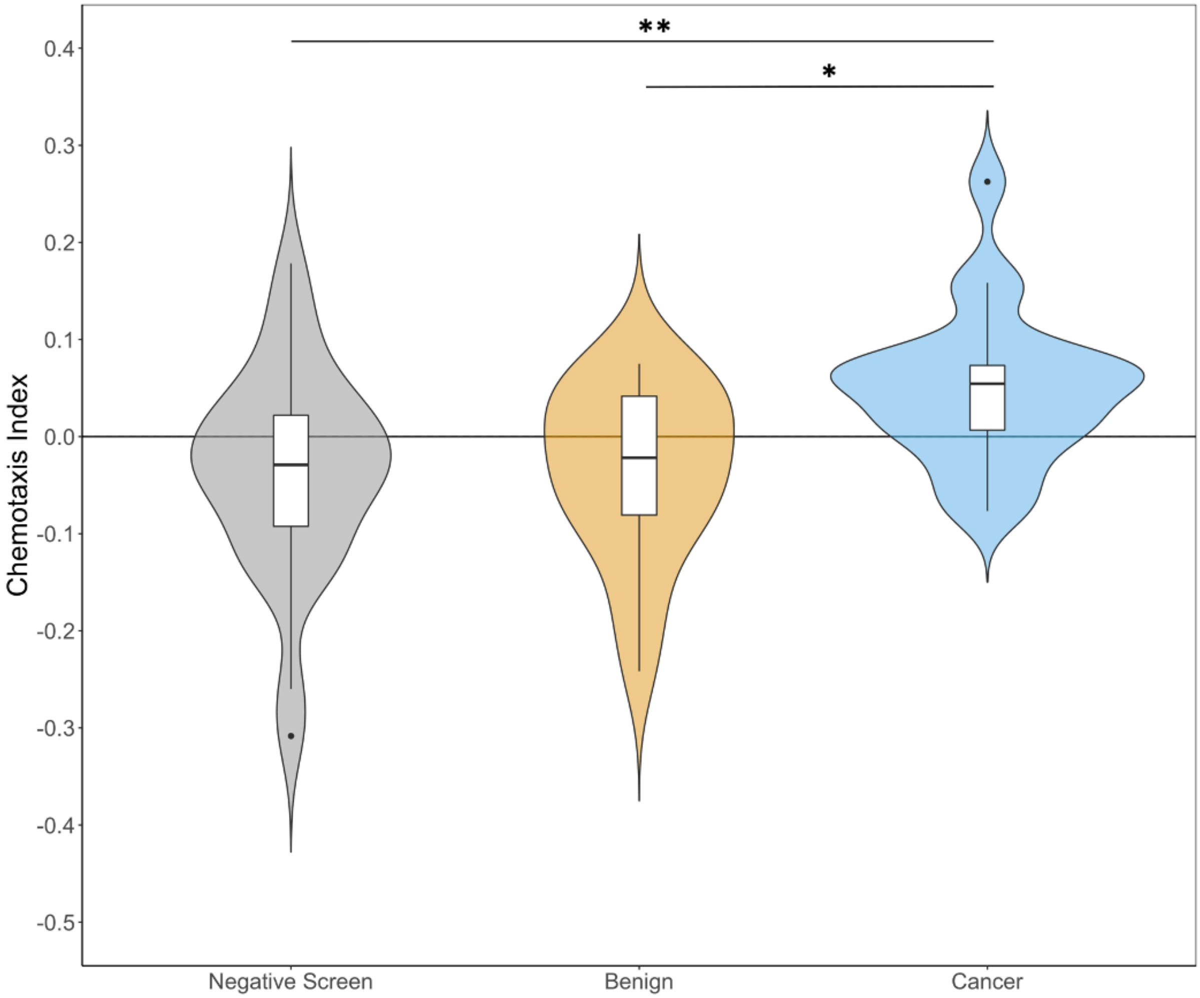

Figure S1. When all technical assay replicates are considered for each sample, *C. elegans* N2 are more attracted to urine from PrCa patients than to benign or negative screen patient urine. Chemotaxis assay technical replicates (n = 4-30 per individual patient) were averaged for negative screen (n = 27 individuals), benign (n = 19 individuals), and cancer (n = 21 individuals) patient urine samples prior to plotting and statistical testing. A Tukey's HSD post hoc test indicated that average CIs for PrCa were significantly higher than those of benign (P = 0.015) and negative screen (P = 0.0083), while benign and negative screen types were not significantly different (P = 0.999). Boxplots for each group are inset within their respective violin plots. *, p < 0.05; **, p < 0.01; ns, not significant.

Biology Open • Supplementary information

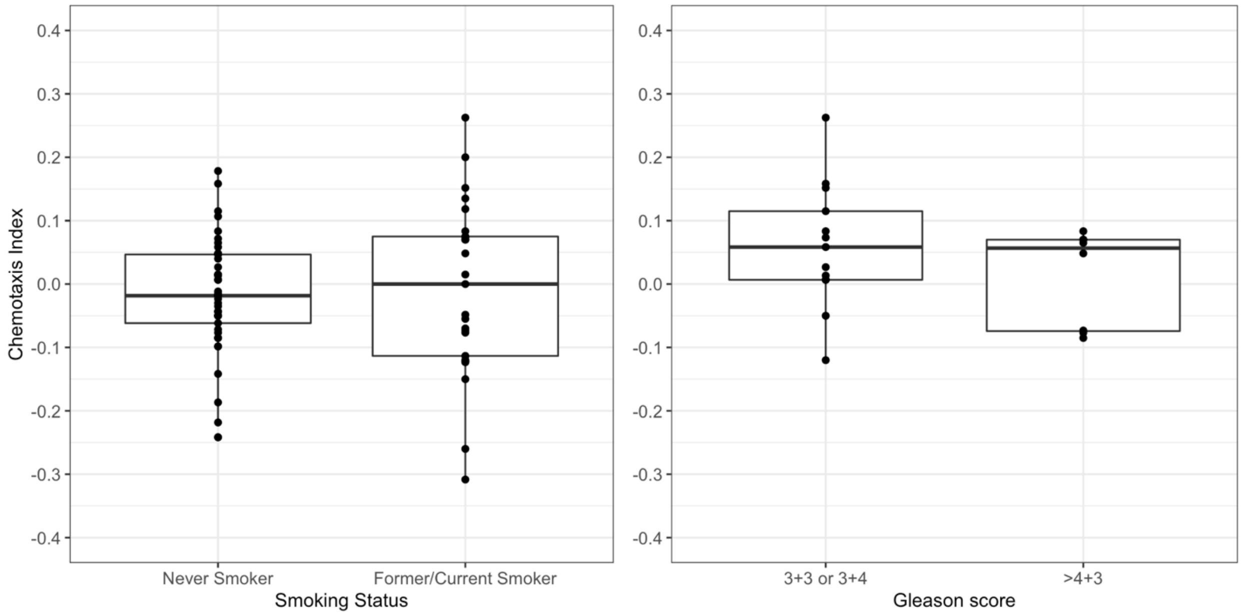

Figure S3. *C. elegans* N2 do not show differences in attraction to urine samples depending on donor smoking status or Gleason score. There was no significant difference in attraction when individuals without a history of smoking were compared to current or former smokers (left panel). Similarly, there was no significant difference in attraction to urine from patients with tumors confirmed to have low (3+3 or 3+4) or high (>4+3) Gleason scores (right panel).

Biology Open • Supplementary information

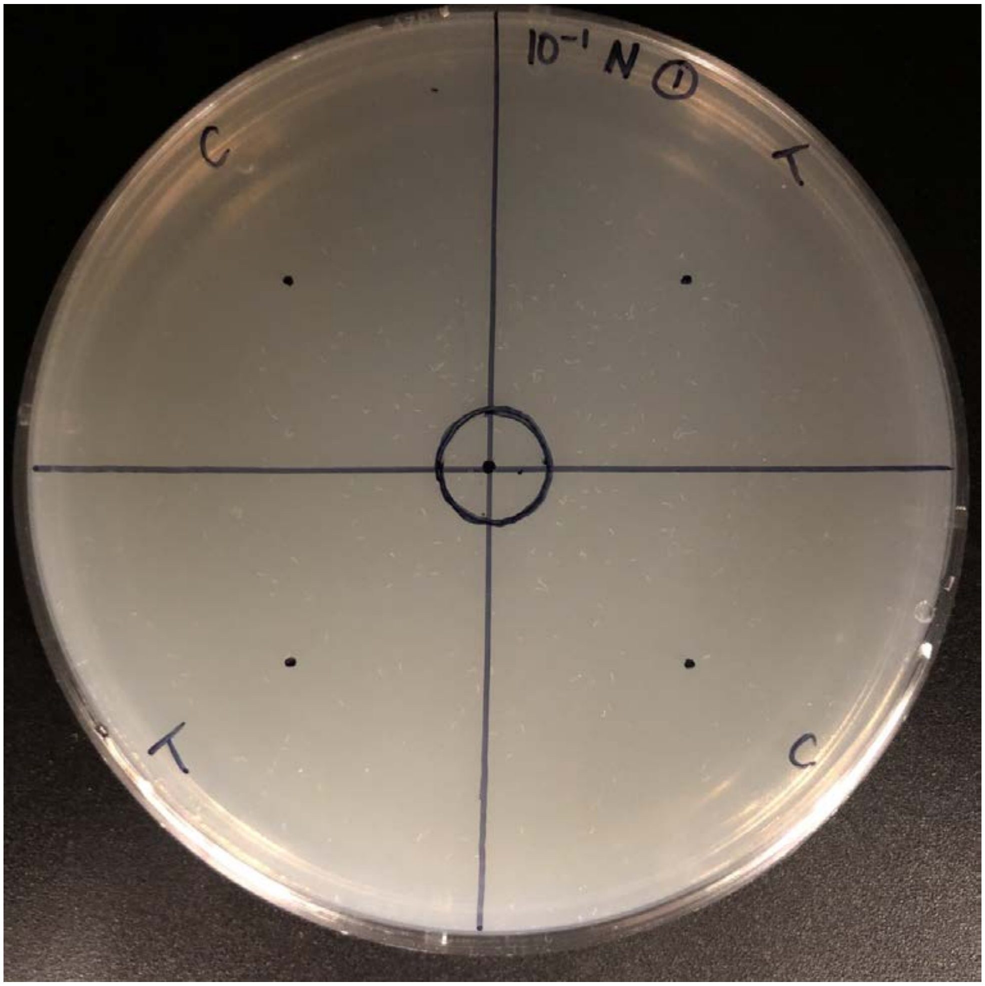

Figure S3. Photograph of a completed chemotaxis assay. Young adult N2 worms were transferred to the center circle and allowed to migrate for 1 hr. Sample (T quadrants) or diluent alone (C quadrants) were spotted on the marked dots.

Biology Open • Supplementary information
